# Reduktion und Absetzen von Antipsychotika in der ambulanten Praxis: Perspektiven von Psychiater*innen

**DOI:** 10.1007/s00115-025-01922-7

**Published:** 2025-11-14

**Authors:** Guillermo Ruiz-Pérez, Nastja Ulrich, Joshua Bettin, Julia Lippert, Thomas Koch, Sebastian von Peter

**Affiliations:** Zentrum für seelische Gesundheit, Abteilung für Psychiatrie und Psychotherapie, Immanuel Klinik Rüdersdorf, Universitätsklinikum der Medizinischen Hochschule Brandenburg, Seebad 82/83, 15562 Rüdersdorf, Deutschland

**Keywords:** Absetzen, Reduzieren, Neuroleptika, Schizophrenie, Shared decision-making, Discontinuation, Reduction, Neuroleptics, Schizophrenia, Shared decision-making

## Abstract

**Hintergrund:**

Antipsychotika (AP) sind ein zentraler Bestandteil der Behandlung psychotischer Störungen. Dabei stellt sich die Frage nach dem Verhältnis zwischen einer langfristigen medikamentösen Behandlung und Möglichkeiten des Reduzierens und des Absetzens (R&A). Die S3-Leitlinie „Schizophrenie“ definiert einen orientierenden Rahmen zum R&A, doch es fehlen bislang konkrete Empfehlungen zur praktischen Umsetzung.

**Ziel der Arbeit:**

Die Einstellungen, Herausforderungen sowie die Reduktionsstrategien ambulanter Psychiater*innen in Berlin und Brandenburg bezüglich dem R&A von AP mittels einer qualitativen Studie zu erforschen, um Rückschlüsse auf den Versorgungsstand und mögliche Defizite zu ziehen.

**Material und Methoden:**

Es wurden zehn leitfadengestützten Interviews mit ambulanten Psychiater*innen sowie eine Fokusgruppe mit Psychiater*innen durchgeführt. Die Daten wurden anschließend mittels thematischer Analyse ausgewertet.

**Ergebnisse:**

Die befragten Psychiater*innen bevorzugen überwiegend eine individuell angepasste, schrittweise Reduktion von Antipsychotika. Während sich einige dabei sicher fühlen und auf eigene Erfahrungswerte zurückgreifen, äußern andere erhebliche Unsicherheiten und Sorgen im Absetzprozess. Diese entstehen teils aus dem Gefühl, bei fehlenden konkreten Vorgaben in der praktischen Umsetzung stark auf sich allein gestellt zu sein. Strukturelle Hürden – wie fehlende psychosoziale Unterstützungsangebote sowie die begrenzte Verfügbarkeit kleinschrittiger Dosierungen – erschweren zudem die Umsetzung wirksamer Reduktionsstrategien.

**Diskussion:**

Eine klarere Positionierung der Leitlinien mit konkreten Empfehlungen zum R&A könnte manche Unsicherheiten der Psychiater*innen abbauen. Mehr psychosoziale und psychotherapeutische Angebote wären notwendig, um den sicheren Prozess des R&A von AP in der ambulanten Praxis zu ermöglichen.

**Zusatzmaterial online:**

Zusätzliche Informationen sind in der Online-Version dieses Artikels (https://doi.org/10.1007/s00115-025-01922-7) enthalten.

## Abstract (en)

**Background:**

Antipsychotic medications (AP) are a central component in the treatment of psychotic disorders. The question arises how to balance long-term pharmacological treatment with the possibility of reduction or discontinuation (R&D). The German S3 guidelines for schizophrenia provide a general framework for R&D but concrete recommendations for practical implementation are still lacking.

**Objective:**

This qualitative study aims to explore the attitudes, challenges and reduction strategies of outpatient psychiatrists in Berlin and Brandenburg regarding the R&D of antipsychotics, in order to draw conclusions about the current state of care and potential gaps in service provision.

**Material and methods:**

In this study 10 semi-structured interviews with outpatient psychiatrists and one focus group with psychiatrists were conducted. The data were analyzed using thematic analysis.

**Results:**

The participating psychiatrists predominantly favored an individualized and gradual approach to reducing antipsychotics. While some reported feeling confident and drew on their own clinical experience, others expressed considerable uncertainty and concern regarding the discontinuation process. This uncertainty was partly attributed to a perceived lack of specific guidance for practical implementation, which led to a sense of individual responsibility. Structural barriers, such as limited psychosocial support services and the lack of availability of finely graduated dosage forms, further hinder the implementation of effective reduction strategies.

**Discussion:**

A clearer position in clinical guidelines, including more specific recommendations on R&D, could help reduce uncertainty among psychiatrists. Furthermore, enhanced psychosocial and psychotherapeutic support services would be necessary to enable a safe R&D process in outpatient settings.

## Hintergrund

Antipsychotika (AP) werden vorrangig zur Behandlung psychotischer Störungen eingesetzt und haben sich in der Akutbehandlung als effektiv erwiesen [1]. Gleichzeitig ist die Einnahme von AP mit erheblichen unerwünschten Wirkungen verbunden, die kurz- und langfristig die Lebensqualität der Betroffenen beeinträchtigen können [2, 3]. In der Praxis stellt sich die Frage nach der optimalen Behandlungsdauer von AP, und damit verbunden, Herausforderungen im Zusammenhang mit dem Reduzieren und Absetzen (R&A). Die Dauer einer antipsychotischen Behandlung zur Rückfallprophylaxe wird in der Fachliteratur unterschiedlich gewichtet. Auch die S3-Leitlinie „Schizophrenie“ verweist auf intensive Diskussionen zu diesem Thema im Rahmen ihrer Erstellung. Zahlreiche Studien zeigen, dass eine kontinuierliche Medikation mit einem deutlich geringeren Rückfallrisiko einhergeht [4, 5]. Gleichzeitig weisen andere Arbeiten darauf hin, dass ein sorgfältig begleitetes R&A langfristig positive Effekte auf kognitive Funktionen, subjektives Wohlbefinden und soziale Teilhabe haben kann [6–8]. Im internationalen Vergleich sind die Leitlinien nicht einheitlich, werden aber insgesamt gegenüber R&A offener [9].

Es fehlen klare, evidenzbasierte Strategien für ein sicheres R&A. Viele randomisierte Studien setzen eine abrupte oder zu schnelle Reduktion um, was das Risiko für Rebound-Phänomene erhöht [10]. Ein systematisches Review zeigt, dass rund die Hälfte der Patient*innen Entzugssymptome erlebt [11], was die Gefahr eines missinterpretierten Rückfalls mit sich bringt. Eine schrittweise, hyperbolische Reduktion über einen längeren Zeitraum könnte dieses Risiko verringern [12, 13]. Dennoch mangelt es an standardisierten Leitlinien und klaren klinischen Empfehlungen zur Umsetzung dieser Strategien. Eine aktuelle systematische Analyse zeigte, dass das „deprescribing“ in der Schizophreniebehandlung zwar in der S3-Leitlinie erwähnt wird, jedoch mit eher allgemeinen Empfehlungen [14]. Neben der Leitlinie werden im deutschsprachigen Raum noch weitere Informationen für Patient*innen, Angehörige und Professionelle angeboten: z. B. in der Broschüre der DGSP [15] und in sonstigen Beiträgen von Betroffenen und Professionellen [16, 17].

Vor diesem Hintergrund stellen die Einstellungen von Psychiater*innen eine entscheidende Rolle beim Prozess des R&A dar. Quantitative Studien weisen darauf hin, dass Psychiater*innen einem R&A bei einer Ersterkrankung eher offen gegenüberstehen und bei Patient*innen mit einer gesicherten Schizophreniediagnose oft eine konservativere Haltung einnehmen [18–20]. Qualitative Untersuchungen machen deutlich, dass Unsicherheiten, Verantwortungsbewusstsein und klinische Erfahrungswerte maßgeblich in die Entscheidung zum R&A einfließen [2, 21].

Die vorliegende Studie untersucht die Einstellungen ambulanter Psychiater*innen in Berlin und Brandenburg hinsichtlich des R&A von AP. Der hier präsentierte Teil der Ergebnisse konzentriert sich auf die praktischen Behandlungsstrategien sowie die Bedürfnisse ambulanter Psychiater*innen, um das R&A in der klinischen Praxis häufiger und sicherer umzusetzen.

## Methoden

### Studiendesign und Rekrutierung

Die Datenerhebung erfolgte über zehn leitfadengestützte Einzelinterviews und eine Fokusgruppe. Die Teilnehmenden wurden über das Register der Kassenärztlichen Vereinigung Berlin-Brandenburg kontaktiert. Von 193 angeschriebenen Psychiater*innen nahmen zehn an Einzelinterviews teil. Ergänzend wurde eine Fokusgruppe mit fünf Psychiater*innen aus psychiatrischen Ambulanzen gebildet. Alle Teilnehmenden gaben eine schriftliche Einverständniserklärung ab. Die Studie wurde von der Ethikkommission der Medizinischen Hochschule Brandenburg genehmigt (Ethikvotum Nr. E‑01-20230224).

### Datenerhebung

Die Interviews und die Fokusgruppe fanden zwischen Juni 2023 und März 2024 statt. Die durchschnittliche Dauer für Einzelinterviews betrug 50 min und 90 min für die Fokusgruppe. Die Gespräche wurden aufgezeichnet, transkribiert und anonymisiert. Der Interviewleitfaden umfasste vier thematische Bereiche zum R&A von AP (s. elektronisches Zusatzmaterial – Interviewleitfaden). Für die Fokusgruppe wurde den Teilnehmenden im Vorfeld repräsentative Zitate aus dem Interviewmaterial zur Verfügung gestellt. Ihre Durchführung erfolgte mithilfe eines spezifischen Leitfadens (s. elektronisches Zusatzmaterial – Fokusgruppeleitfaden), bei dem die Fragen sich auf Zitate, welche den Teilnehmenden geschickt wurden, bezogen.

### Datenanalyse

Die Analyse der Transkripte erfolgte mittels einer induktiven, modifizierten thematischen Analyse mit der Software MAXQDA (VERBI-Software GmbH, Berlin, Deutschland) [22]. Die Transkripte wurden in mehreren Kodierungsrunden von drei Forschenden unabhängig voneinander analysiert und in einer Konsensdiskussion zu einer finalen Struktur zusammengeführt. Die Ergebnisse wurden iterativ überprüft, um methodische Qualität und Kohärenz sicherzustellen.

## Ergebnisse

### Übliche Absetz- und Reduktionsstrategien

#### Vorbereitende Maßnahmen

Zur erfolgreichen Umsetzung des R&A nannten die Befragten verschiedene Schritte, darunter die informierte Zustimmung der Patient*innen sowie Strategien zur Früherkennung möglicher Symptome. Konkret wurde die Erkennung von Frühwarnhinweisen bei Verschlechterung, erhöhte Verfügbarkeit ambulanter Dienste, Einbeziehung des sozialen Netzwerks der Patient*innen und Erstellung eines Notfallplans empfohlen. Eine Stimme betonte: „Gute Aufklärung und Vorbereitung und eine enge Begleitung können Ängste reduzieren und den Erfolg eines Absetzens erheblich verbessern“ (I-1).

#### Technische Umsetzung

Die Mehrheit sprach sich für eine langsame Reduktion mit zunächst größeren Schritten, anschließend stark verfeinerten ggf. individuellen Anpassungen aus. Dies sorge auch bei den Ärzt*innen für Sicherheit: „Ein schrittweises Vorgehen hilft nicht nur den Patient*innen, sondern gibt auch den Behandler*innen mehr Sicherheit im Umgang mit dem Prozess“ (I-3). Begleitmaßnahmen wie engmaschige Nachsorge und psychotherapeutische Unterstützung wurden als zentral betrachtet. Ein Befragter betonte: „Man kann nicht einfach die Medikation reduzieren und abwarten, sondern muss eng mit den Patient*innen zusammenarbeiten, um frühzeitig auf Veränderungen reagieren zu können“ (FGP).

### Erlebte Herausforderungen

#### Suche nach der minimal effektiven Dosis und Monotherapie als Ziel

Viele Psychiater*innen betonten die Bedeutung einer minimal effektiven Dosis, um Nebenwirkungen zu reduzieren und dennoch ein erhöhtes Rückfallrisiko zu vermeiden. „Das Ziel sollte sein, die Patienten mit der geringstmöglichen Dosis zu stabilisieren und, wenn möglich, ganz auf die Medikation zu verzichten“ (FGP). Die Mehrheit der Interviewten empfand eine langfristige Therapie mit kleinen Dosierungen als sicherer gegenüber dem Absetzen. Ein weiteres Ziel sei die Vermeidung von Polypharmazie und hohen Dosierungen.

#### Die schwierige Entscheidung zwischen dem Absetzen und der Langzeittherapie

Die teilnehmenden Psychiater*innen äußerten unterschiedliche Meinungen über die angemessene Dauer der AP-Behandlung. Einige befürworteten eine langfristige Medikation zur Rückfallprävention, während andere eher ein frühzeitiges Absetzen wagen würden. Eine der Widerstände ist die Unsicherheit, die manche Psychiater*innen dabei erleben. Eine Teilnehmerin fasste diese zusammen: „Manchmal habe ich das Gefühl, dass wir die Patienten zu lange auf der gleichen Medikation halten, weil wir Angst haben, etwas zu verändern“ (FGP). Gleichzeitig wiesen einige darauf hin, dass es sehr individuell sei, den richtigen Zeitpunkt für ein Absetzen zu erkennen.

#### Persönliche und äußere Widerstände

Trotz einer allgemein positiven Haltung gegenüber dem R&A beschrieben viele Psychiater*innen die Sorge, bei einer möglichen Verschlechterung zur Rechenschaft gezogen zu werden. Eine Befragte berichtete: „Die größte Angst ist nicht unbedingt der Rückfall selbst, sondern die Schuld, die man als Behandler empfindet, wenn es passiert“ (FGP). Ein weiterer Aspekt ist hierbei der Druck von Angehörigen, die eine Reduktion oft ablehnen, da sie eine Verschlechterung befürchten: „Das Gefühl, dass Angehörige oder Kollegen einen für einen Rückfall verantwortlich machen könnten, ist oft ein entscheidender Faktor dafür“ (I-8).

### Bedarf an sicherem R&A

#### Klarere Leitlinienempfehlungen

Ein wiederkehrendes Thema war der Wunsch nach einheitlichen, klaren Empfehlungen. Mehrere Psychiater*innen bemängelten, dass konkrete Leitlinien fehlen. Eine Psychiater*in meinte: „Wenn die Leitlinien klarere Empfehlungen geben würden, wäre es für uns einfacher, das Absetzen systematisch anzugehen“ (FGP). Das würde dazu beitragen, dass die Hauptlast der Entscheidung nicht größtenteils bei jeder einzelnen Psychiater*in liegt, sondern dass die offiziellen Leitlinienempfehlungen ihr Handeln stützen würden.

#### Verfügbarkeit kleiner Dosierungen und individuelle Strategien

Wiederholt wurde das Problem genannt, dass nicht alle AP in feinen Dosierungen verfügbar seien und mehrfach erklärt, dass es einfacher sei, wenn es mehr Optionen für geringe Dosierungen gäbe. Eine Psychiater*in erklärte: „Die Reduktion muss individuell angepasst werden, aber oft fehlen uns die Möglichkeiten, das in der Praxis optimal umzusetzen“ (FGP).

#### Forderung nach mehr Psychotherapie und medikamentenfreien Therapien

Einige Befragte äußerten den Wunsch nach einer stärkeren Einbindung psychotherapeutischer Verfahren und weiterer nichtmedikamentöser Angebote, um die Häufigkeit einer erfolgreichen Reduktion zu erhöhen. „Psychotherapie spart Medikamente“ (FGP), lautete eine Zusammenfassung. Außerdem hieß es: „Mehr therapeutische Angebote würden uns helfen, Patient*innen besser zu unterstützen, ohne direkt auf Medikamente zurückgreifen zu müssen“ (I-3). So bekräftigten viele Interviewte das Desideratum, dass es für Patient*innen mit Psychosen im ambulanten Setting mehr Ressourcen für psychotherapeutische Therapieangebote geben sollte.

## Diskussion

Die vorliegende Studie zeigt, dass ambulante Psychiater*innen sich beim R&A von Antipsychotika in einem Spannungsfeld zwischen Rückfallprophylaxe und Patientenautonomie bewegen. Viele schildern eine ausgeprägte Sorge, im Fall einer Verschlechterung verantwortlich gemacht zu werden, sowie sozialen Druck durch Angehörige. Die minimale effektive Dosierung wird häufig als pragmatischer Kompromiss zwischen Absetzen und Erhaltungstherapie verstanden. Darüber hinaus betonten mehrere Teilnehmende, dass ihre Haltung zum R&A stark vom Krankheitsverlauf abhängt: Während beim Vorliegen einer Ersterkrankung Offenheit besteht, wird bei mehrfachen Episoden meist eine Fortführung der Medikation bevorzugt. Zudem wurden konkrete Maßnahmen genannt, um Risiken beim R&A zu minimieren – etwa Frühwarnzeichen, Notfallpläne oder die Einbindung des sozialen Umfelds. Ein wiederkehrendes Anliegen war der Wunsch nach klareren, verbindlicheren Leitlinien, die die individuelle Verantwortung entlasten könnten.

Diese Ergebnisse ergänzen, präzisieren und erweitern bestehende Forschung. Studien haben wiederholt auf das erhöhte Rückfallrisiko bei abruptem Absetzen [4, 5], mögliche Vorteile einer begleiteten Reduktion [6–8] sowie die Problematik zu schneller Reduktionsprotokolle hingewiesen [10, 11]. Die Relevanz langsamer, hyperbolischer Strategien ist bekannt [12, 13], wird in der Praxis jedoch durch fehlende kleinteilige Dosierungen vieler Präparate eingeschränkt [14]. Auch der in der Literatur beschriebene Einfluss klinischer Erfahrung und Verantwortung auf Entscheidungen [2, 21] wird durch unsere Studie bestätigt.

### Die Praxis des R&A im Licht der aktuellen S3-Leitlinie

Die S3-Leitlinie „Schizophrenie“ wurde bereits als fortschrittlich in Bezug auf das Thema des R&A von AP eingeschätzt [23, 24]. Die Teilnehmenden stimmten mit mehreren ihrer zentralen Empfehlungen überein. Besonders deutlich wird dies in Bezug auf die Empfehlung der individuellen Anpassung der AP-Dosis und die Bedeutung einer schrittweisen Dosisreduktion, um Rezidive zu vermeiden.

Ein weiteres zentrales Thema ist die Bedeutung psychosozialer Unterstützungsangebote im Absetzprozess. Die befragten Psychiater*innen schätzten begleitende psychotherapeutische und soziale Maßnahmen als essenziell für eine erfolgreiche Reduktion ein. Das geringe ambulante Psychotherapieangebot für Patient*innen mit Psychosen wurde von den Teilnehmenden als sehr problematisch betrachtet. Die S3-Leitlinie „Schizophrenie“ fordert eine engmaschige Begleitung bspw. durch multiprofessionelle Teams und schließt explizit psychosoziale Maßnahmen als Teil der Standardbehandlung mit ein. In der Praxis scheinen diese Angebote nicht ausreichend verfügbar zu sein. Dies erschwert eine sichere Umsetzung des R&A erheblich und führt zur Fortsetzung der Medikation als Behandlungskonzept.

Es gibt auch Abweichungen zwischen diesen Studienergebnissen und den Leitlinienempfehlungen. Während die befragten Psychiater*innen eine Reduktion insbesondere bei Patient*innen mit einer ersten Episode unterstützen, tendieren sie bei mehrfach erkrankten Patient*innen zu einer Erhaltungstherapie im Sinne einer Rezidivprophylaxe. Die S3-Leitlinie gibt keine Empfehlung zur Behandlungsdauer, sondern belässt die Entscheidung einer individuellen Risiko-Nutzen-Abwägung den behandelnden Ärzt*innen und ihren Patient*innen. Während frühere Versionen empfohlene Zeiträume für die Erhaltungstherapie vorgaben [25], wurde in der aktuellen Version auf solche Vorgaben verzichtet. Jedoch wird auf ein erhöhtes Rezidivrisiko im Fall einer R&A hingewiesen. In der Praxis scheint die Unterscheidung zwischen einer und multiplen Episoden aber entscheidend zu sein, sodass die Mehrheit der Teilnehmenden sich im Fall der zweiten bzw. dritten Episode oder der bestätigten Diagnose einer Schizophrenie für eine Dauertherapie entscheiden.

### Konsequenzen für den klinischen Alltag

Ein zentrales Ergebnis dieser Studie ist die Unsicherheit, die viele ambulante Psychiater*innen beim R&A empfinden. Diese resultiert nicht nur aus fehlenden individuellen Erfahrungswerten, sondern ist eng mit der unzureichenden Evidenzlage verknüpft. Außerdem äußerten viele die Sorge, dass sie für ein Rezidiv verantwortlich gemacht werden könnten.

Die S3-Leitlinie adressiert diese Unsicherheit nicht direkt, sondern verweist auf eine gemeinsame Entscheidungsfindung. Während dies theoretisch eine Entlastung für Psychiater*innen darstellen könnte, scheint es in der Praxis nicht auszureichen, um die empfundene Verantwortung zu reduzieren. Fehlt es an konkreten, über die in der S3-Leitlinie bereits enthaltenen Grundideen für eine Dosisreduktion hinausgehenden Leitlinienvorgaben, sind die Psychiater*innen dazu gezwungen, die eher allgemeinen Empfehlungen für sich weiter zu konkretisieren. Das macht ihre persönliche Haltung zum entscheidenden Faktor. Das sorgt in der Praxis für heterogene Behandlungskonzepte: Während einige Psychiater*innen ein hohes Maß an Offenheit gegenüber Absetzversuchen zeigen, tendieren andere dazu, ein chronisches Konzept der psychotischen Diagnose zu haben und das R&A abzulehnen bzw. sich kritisch demgegenüber zu äußern. Die Entscheidung, Patient*innen beim R&A von AP zu unterstützen, wird demnach individuell durch die Behandler*innen gefällt. Dadurch verbleibt die Hauptlast der Verantwortung bei den Psychiater*innen, was die beschriebenen Sorgen und Unsicherheiten und demzufolge ein konservatives Behandlungskonzept verstärkt, wie auch andere Studien zeigen konnten [2, 21].

In Deutschland finden sich, außerhalb der Leitlinien, konkretere Empfehlungen: Ein relevantes Beispiel dafür ist die Broschüre der DGSP, die länger als ein Jahrzehnt besteht und regelmäßig aktualisiert wird [15]. Andere Literaturquellen, die sowohl für Patient*innen als auch Angehörige und Professionelle konzipiert sind, enthalten Praxisempfehlungen für das R&A von Psychopharmaka generell und von AP im Speziellen [16, 17]. Auf internationaler Ebene finden sich wissenschaftliche Artikel, die praktische Strategien zum R&A darstellen [10, 24, 26]. Die Empfehlungen für das Absetzen von Antidepressiva sind dabei im Vergleich deutlich verbreiteter und systematischer ausgearbeitet (u. a. [28, 29]). Beispielsweise wurden in der NICE-Guideline „Medicines associated with dependence or withdrawal symptoms“ Antidepressiva aufgenommen [29], AP hingegen nicht berücksichtigt [23].

Obwohl es immer mehr Erkenntnisse darüber gibt, wie das Absetzen am besten gestaltet werden könnte, bleiben Leitlinien der zentrale Orientierungsrahmen für verschreibende Ärzt*innen. Klarere Empfehlungen in der Leitlinie könnten dabei helfen, diese Unsicherheiten abzubauen, das Vertrauen in Absetzstrategien zu stärken und langfristig mehr Offenheit gegenüber dem R&A zu fördern.

## Fazit

Die vorliegenden Ergebnisse weisen darauf hin, dass ambulant tätige Psychiater*innen bei dem R&A von AP mit strukturellen und prozessbezogenen Herausforderungen konfrontiert sein können. Dazu zählen insbesondere die als unzureichend konkret wahrgenommenen Leitlinienempfehlungen sowie Rahmenbedingungen, die ein differenziertes und individuell angepasstes R&A erschweren könnten. Mehrere Befragte berichteten von Unsicherheiten und einem ausgeprägten Verantwortungsgefühl, das sie im Umgang mit potenziellen Rückfällen belastet. Zudem wurde der Mangel an psychosozialen und psychotherapeutischen Unterstützungsangeboten im ambulanten Setting als hinderlich beschrieben. Vor diesem Hintergrund erscheint eine stärkere Ausdifferenzierung leitlinienbasierter Empfehlungen sowie der Ausbau begleitender, nichtmedikamentöser Versorgungsangebote als mögliche Ansatzpunkte zur Unterstützung einer besseren Praxis beim R&A.

## Supplementary Information

Fokusgruppeleitfaden

Interviewleitfaden

Zusatzmaterial Soziodemographie

## Data Availability

Die erhobenen Datensätze können auf begründete Anfrage in anonymisierter Form beim korrespondierenden Autor angefordert werden.

## Abkürzungen

AP: Antipsychotika
DGSP: Deutsche Gesellschaft für Soziale Psychiatrie
FGP: Fokusgruppe mit Psychiater*innen
I‑x: Einzelinterview
NICE: National Institute for Health and Care Excellence UK
R&A: Reduzieren und Absetzen
