# Supplementary material for: Reduction and discontinuation of antipsychotic drugs in outpatient practice: perspectives of psychiatrists
Source: Nervenarzt. 2025 Nov 14;97(5):468–73. [Article in German] doi: 10.1007/s00115-025-01922-7 (PMC13550059; doi:10.1007/s00115-025-01922-7)
Supplement: Supplementary file 1 [file 115_2025_1922_MOESM1_ESM.docx]

Fokusgruppeleitfaden — TaNEA

1. Wie stehen Sie überhaupt zum Thema Absetzen von NL? Und zur Erhaltungstherapie/Rezidivprophylaxe?

„Und da ist es wichtig, das einfach rational im Auge zu behalten und eben mit Abklingen eines akuten psychotischen Schubes dann auch wieder mit der Medikation runterzugehen.“

„Und ja, könnte man sagen, also mindestens ein Jahr und dann muss man sehen, ob man das dann eben auch weiter sogar fortführt. Definitiv.“

„Viele verstehen ja auch, also viele, die schwer erkrankt gewesen sind, die verstehen ja auch, dass sie für ihre Remission auch einen gewissen Preis zahlen müssen.“

„Also Ausgangshaltung ist schon eher langfristige Rezidivprophylaxe. Und wenn ich dann den Eindruck habe, das könnte auch ohne gehen, dann würde ich das vorschlagen.“

„Was ich erlebt habe über die Jahre, wenn die handfeste F20er-Diagnosen hatten, am ehesten brauchten die immer wieder Medikamente“

„Ja, also mein Wissen darüber, wenn es da wirklich in der Schizophrenie ist, wird es ohne Medikamente nicht gehen.“

Fallvignette

„A: Und in dem Fall, wie würden Sie reagieren? Um bei diesem Beispiel zu bleiben. Dieser junge Mann, sechs Wochen schon raus aus der Klinik, noch mit Kombinationstherapie, und er sagt: "Ich will eigentlich keine Medikamente mehr. Wie können wir es irgendwie umgesetzt kriegen?" Sie würden natürlich empfehlen, es weiter zu nehmen, wie Sie vorhin gesagt haben. Aber wenn die Person darauf besteht, wie oft auch der Fall ist.

B: Also letztendlich akzeptiere ich es. Bleibt einem ja auch nichts weiter übrig. Weil ich bitte, dass er trotzdem zu mir kommt. Also ich sage: "Okay, passen Sie auf, ich kann jetzt nichts machen. Das wissen Sie ja, das weiß ich. Aber bitte kommen Sie trotzdem weiter zu mir." Und das passiert dann manchmal ja, manchmal nicht. Aber das ist immer mein Angebot, dass Sie von mir aus... dann sollen Sie die Pillen absetzen, kann sowieso nichts gegen machen. Aber dass wir zumindest in Beziehung bleiben können und in Kontakt und wir trotzdem die Behandlung weiterführen.

A: Und würden Sie die Person unterstützen bei dem Absetzen? Oder würden Sie einfach sagen, dann setzen Sie das ab?

B: Also ehrlich gesagt, da würde ich die Person in dem ganz konkreten Fallbeispiel ehrlich gesagt nicht unterstützen. Aber wie es so ist, wenn man nichts tun kann, dann will man zumindest das Angebot machen, dass deshalb nicht die Behandlung enden muss. Also ich mache keinen Beziehungsabbruch, nur weil jemand nicht macht, was ich will. Dann wäre ich nicht mehr in Partnerschaft und hätte keine Kinder. Also man kann das, was man will, nicht durchsetzen, aber die Beziehung kündige ich nicht auf.“

2. Wie geht es Ihnen bei Absetzprozessen? Welche Schwierigkeiten erleben Sie?

„Also in den meisten Fällen ist das für mich neutral. Also das ist einer von den Wünschen, die an mich angetragen werden. Dann überlege ich, wie man mit dem Wunsch umgehen kann, wie man ihn erfüllen kann. Es gibt halt immer mal ein paar Fälle, wo ich ein sehr schlechtes Gefühl habe, wo ich denke, das sollte der nicht tun. Und das versuche ich denen dann auch zu vermitteln. Manchmal hilft es, manchmal nicht. Und manchmal muss ich mich dann selber auch belehren lassen, dann haben die beim nächsten Mal oder übernächsten Mal eben trotzdem abgesetzt oder haben vorher schon abgesetzt und beichten das jetzt erst oder so und dann geht's trotzdem gut. Das ist eigentlich das Unangenehme, wenn man zwar so Gefühle und Einstellungen hat, aber sich das mit dem tatsächlichen Verlauf oft nicht so richtig deckt, dass man immer wieder Überraschungen erlebt.“

3. Was bräuchten Sie um besser/mehr absetzen zu können?

„B: Ja, es wäre sicherlich hilfreich, wenn es für die verschiedenen Medikamente, die man hat, bei den Dosierungsrichtlinien vielleicht auch mehr Hinweise dazu gäbe, wie setze ich so ein Medikament eigentlich ab. Zum Beispiel ich habe einen Patienten, der ist seit einem halben Jahr eingestellt mit Risperidon in einer bestimmten Dosierung. Wie dosiere ich das sinnvoll runter, in welcher Geschwindigkeit, mit welchen Veränderungsintervallen?“

4. Was spricht für ein erfolgversprechendes Absetzen?

„Ich sage mal eine Abdosierung ist umso einfacher, je besser Patienten psychosozial eingebunden sind. Stichwort Eigengefährdung, Stichwort Krankheitseinsicht oder auch soziale Garanten, die vielleicht ein bisschen mit draufschauen können. Wenn jemand einsam ist und kaum Kontakte hat, dann tut man sich deutlich schwerer, Medikamente abzudosieren, als wenn man weiß, der trifft mindestens alle zwei, drei Tage mal seinen besten Freund oder die Mutter ruft immerzu an oder sowas. Das finde ich einen wichtigen Parameter. Dann Stabilität und Introspektionsfähigkeit vom Patienten. Also Psychosen haben ja auch Vorläufersymptome, fängt ja oft wieder an mit irgendwelchen Wahrnehmungsveränderungen oder leichter Paranoia oder Schlafstörungen, Konzentrationsstörungen. Wenn die Patienten fähig sind, da auch sich selbst einigermaßen einzuschätzen, dann macht es einem das als Behandler auch leichter.“

5. Wie geht man mit Absetzphänomenen und Rezidiven um?

„Ja, also nach meiner Erfahrung treten diese Absetzphänomene vor allem dann auf, wenn ich plötzlich absetze oder zu schnell reduziere. Wenn ich mit der Dosis wirklich langsam runtergehe - die Zeit haben wir ja in der Regel, es sei denn der Patient drängt und sagt, ich will jetzt nicht mehr - nein, das kann man glaube ich ganz gut differenzieren“

„Und bis 200 habe ich gedacht, prima, also da habe ich kein Problem, und dann drängelte sie aber auf immer mehr und bei 100 Milligramm ist sie dann re-exazerbiert. Das ist dann, wenn Sie eine lange Beziehungsarbeit mit jemandem pflegen, auch nicht so ein irres Problem, die Leute dann auf Station zu bringen, wieder mit der Akutmedikation aufzudosieren und dann aus den gemeinsamen Erfahrungen sozusagen die Lehren zu ziehen. Und die bekommt jetzt wieder, glaube ich, eine Tages-Dosis von 300 Milligramm. Mal gucken, sicher für das kommende Jahr und dann muss man weitersehen und dann weiß man, unter 200 sollte man nicht gehen.“

6. Welche Rolle spiel das soziale Umfeld?

„Einfach hier Rezidivgefahr. Ich meine, das ist ja das Problem. Dann weiß man auch nicht, inwiefern Angehörige da irgendwie das wollen, dann gibt es ja auch vielleicht Konflikte. "Du darfst nicht reduzieren, sonst passiert das wieder, dann trenne ich mich." Also psychologische Gründe. Oder auch hier Familienproblematiken, die da so hinter stehen, das ist auch noch nicht ganz selten. Eigenfremdgefährdung, immer ein Thema. Fahrtüchtigkeit, auch ständig ein Thema. Wenn die absetzen und ein Rezidiv kriegen, Vernachlässigung, vielleicht Vermüllung. Wenn die ein Rezidiv kriegen, Wohnungsverlust, können danach irgendwie keine eigene Bleibe mehr haben, Partnerschaftsverlust, was weiß ich“

„Angehörige wollen immer, dass nichts passiert. Angehörige, das ist noch viel schlimmer bei den armen Bipolaren. Der Angehörige soll lieber ein bisschen zu depressiv sein und nicht stören und weiter auffallen, als irgendwie hypoman werden. Kaum sind die Leute mal aus meiner Sicht irgendwie gut drauf oder haben ein gutes Funktionsniveau, dann hat man sozusagen als Nächstes einen Angehörigen am Apparat, der sagt: "Wenn das nicht mal wieder losgeht..." Kann ich auch in guten Grenzen nachvollziehen“
