# Supplementary material for: Reduction and discontinuation of antipsychotic drugs in outpatient practice: perspectives of psychiatrists
Source: Nervenarzt. 2025 Nov 14;97(5):468–73. [Article in German] doi: 10.1007/s00115-025-01922-7 (PMC13550059; doi:10.1007/s00115-025-01922-7)
Supplement: Supplementary file 2 [file 115_2025_1922_MOESM2_ESM.docx]

|  | Übergeordnete Leitfrage: | Konkretisierende Fragen: | Aufrechterhaltungs-/Steuerungsfragen: |
| --- | --- | --- | --- |
| **1** | **Haltung**  - Wie stehen Sie zur Reduktion und Absetzen von Psychopharmaka? | - Glauben Sie, es ist wichtig, im Laufe der Therapie mit Psychopharmaka, diese irgendwann zu reduzieren und abzusetzen? |  |
|  |  | - Haben Sie sich im Laufe Ihrer Karriere mit diesem Thema theoretisch auseinandergesetzt? |  |
|  |  | - Was halten Sie vom Begriff/Konzept der Compliance? |  |
| **2** | **Praxis**  - Welche Erfahrung haben Sie in Ihrer bisherigen Praxis damit gemacht? | - Wird eine Reduktion/ein Absetzen meistens von Ihren PatientInnen angeregt oder durch Sie? | - Welche Gründe geben die PatientInnen meistens an?  - Wie stehen Sie dazu, wenn PatientInnen danach fragen? |
|  |  | - Bei welchen PatientInnen kommt das meistens in Frage?  - Welche Aspekte der Behandlung und der PatientInnen spielen eine Rolle, um sich für eine Reduktion/Absetzung zu entscheiden? (*Zustand, bisheriger Verlauf, NW, Vorgeschichte mit Rezidiven, verfügbare nicht-pharmakologische Therapieangebote, soziale Situation, beruflicher Status…*) | (*Daten der Vorstudien können hier dargestellt werden*)  - Glauben Sie, dass es Unterschiede zwischen Diagnose oder Psychopharmakagruppen gibt?  - Fäll es Ihnen leichter, die Indikation der Reduktion/Absetzung bei manchen Diagnosen bei ähnlicher Krankheitsphase zu stellen? (z.B. einfacher für Depression als für Schizophrenie, selbst wenn in beiden Fällen die Erkrankung remittiert erscheint)  - Fühlen Sie sich einer Reduktion unterschiedlich sicher je nach Medikamenten-/Diagnosengruppe? |
|  |  | - Welche ist Ihre Vorgehensweise, wenn ein Reduktions-/Absetzversuch unternommen wird?  - Wie besprechen Sie es mit den PatientInnen? | - Welche pharmakologischen und nichtpharmakologischen Maßnahmen kommen in Frage?  - Erfolgt eine Aufklärung im Voraus?  - Berücksichtigen Sie die künftige Reduktion und Absetzung bei der Ansetzung von Psychopharkama (z.B. Medikamentenwahl und -dosierungen)? |
|  |  | - Werden Reduktions- und Absetzversuche mit den PatientInnen gemeinsam geplant? (*shared decision making*) | - Werden die PatientInnen bei dem Entwurf der Reduktionsstrategie einbezogen?  - Wird das soziale Netz der PatientInnen auch mit einbezogen? |
| **3** | **Schwierigkeiten/Hindernisse**  - Welche Herausforderungen stellen sich bei einer Reduktion bzw. während des Absetzens von Psychopharmaka? | - Welche Sorgen, Ängste oder Unsicherheiten treiben Sie hier um? | - Erschweren Ihre Sorgen/Ängste die Indikationsstellung einer Reduktion/Absetzung?  - Glauben Sie, dass Sie über ausreichendes Wissen bzw. genügend Erfahrungen in dem Feld verfügen?  - Haben Sie sich zu dem Thema ausgebildet/haben Sie Fortbildungen (bspw. DGPPN, Fachliteratur…) besucht? |
|  |  | - Wie gehen Sie mit Rezidiven und Absetzphänomenen um? | - Welche Schwierigkeiten haben Sie in solchen Fällen?  - Besprechen Sie es mit Ihren PatientInnen im Voraus, dass solche Situationen auftreten können? |
|  |  | - Welche strukturellen Hindernisse gibt es, die ein Absetzen oder die Reduktion von Psychopharmaka behindern? (*Struktur der psychiatrischen ambulanten Versorgung, Finanzierung, nicht-medikamentöse Therapieangebote, Haltung der Institution und Vorgesetzen dazu…*) | - Gibt es externe Faktoren, die Sie verhindern, mehr/häufiger Reduktions- und Absetzungsversuche unternehmen? |
| **4** | **Bedürfnisse**  - Was bräuchten Sie, um mehr Reduktionen und Absetzung bzw. diese besser durchführen zu können? | - Welches Wissen / Welche Instrumente bräuchten Sie, um sich dabei sicherer fühlen? | - Was könnten Sie dafür tun?  - Welche Maßnahmen seitens der Institutionen wären dafür sinnvoll? (*Krankenkassen, Fortbildungen von der Ärztekammer, Pharmakonzerne —Tapering-taugliche Präparate—etc…*) |
|  |  | - Was würden Sie sich wünschen, um Ihren PatientInnen dabei besser helfen/unterstützen zu können? | - Sind Ihre Arbeitsbedingungen/Ihr Arbeitsrahmen dafür gut geeignet? Was sollte sich dabei ändern?  - Welche begleitende Therapiemaßnahmen wären für Sie wichtig, um erfolgreiche Reduktions- und Absetzversuche zu erreichen? |
