# Supplementary material for: Reduction and discontinuation of antipsychotic drugs in outpatient practice: perspectives of psychiatrists
Source: Nervenarzt. 2025 Nov 14;97(5):468–73. [Article in German] doi: 10.1007/s00115-025-01922-7 (PMC13550059; doi:10.1007/s00115-025-01922-7)
Supplement: Supplementary file 3 [file 115_2025_1922_MOESM3_ESM.docx]

| Interviews | n = 10 |  |  |
| --- | --- | --- | --- |
| Geschlecht | m | w |  |
|  | 5 | 5 |  |
| Alter | Durschnitt |  |  |
|  | 52,7 | (40 — 65) |  |
| ambulante Erfahrung (in Jahren) | < 10 | 10 — 20 | >20 |
|  | 4 | 4 | 2 |
|  |  |  |  |
| Fokusgruppe mit Psychiater*innen | n = 5 |  |  |
| Geschlecht | m | w |  |
|  | 4 | 1 |  |
| Alter | Durschnitt |  |  |
|  | 41,4 | (40 — 47) |  |
| ambulante Erfahrung (in Jahren) | < 10 | 10 — 20 | >20 |
|  | 3 | 2 | 0 |
